# An intermediate crocodylian linking two extant gharials from the Bronze Age of China and its human-induced extinction

**DOI:** 10.1098/rspb.2022.0085

**Published:** 2022-03-09

**Authors:** Masaya Iijima, Yu Qiao, Wenbin Lin, Youjie Peng, Minoru Yoneda, Jun Liu

**Affiliations:** ^1^ School of Resource and Environmental Engineering, Hefei University of Technology, 193 Tunxi Road, Baohe, Hefei, Anhui 230009, People's Republic of China; ^2^ Department of Biological Sciences, Clemson University, Clemson, SC 29634, USA; ^3^ Nagoya University Museum, Furocho, Chikusa-Ku, Nagoya, Aichi 464-8601, Japan; ^4^ Xinhui Museum, 12 Gongyuan Road, Xinhui, Jiangmen, Guangdong 529199, People's Republic of China; ^5^ Shunde Museum, Bishui Road, Shunde, Foshan, Guangdong 528300, People's Republic of China; ^6^ The University Museum, The University of Tokyo, Hongo 7-3-1, Bunkyo, Tokyo 113-0033, Japan

**Keywords:** phylogeny, crocodylia, acoustics, sexual selection, extinction, Holocene

## Abstract

A solid phylogenetic framework is the basis of biological studies, yet higher level relationships are still unresolved in some major vertebrate lineages. One such group is Crocodylia, where the branching pattern of three major families (Alligatoridae, Crocodylidae and Gavialidae) has been disputed over decades due to the uncertain relationship of two slender-snouted lineages, gavialines and tomistomines. Here, we report a bizarre crocodylian from the Bronze Age of China, which shows a mosaic of gavialine and tomistomine features across the skeleton, rendering support to their sister taxon relationship as molecular works have consistently postulated. Gavialine characters of the new Chinese crocodylian include a novel configuration of the pterygoid bulla, a vocal structure known in mature male Indian gharials. Extinct gavialines have repeatedly evolved potentially male-only acoustic apparatus of various shapes, illuminating the deep history of sexual selection on acoustic signalling in a slender-snouted group of crocodylians. Lastly, a cutmark analysis combined with accelerator mass spectrometry (AMS) radiocarbon dating of bone remains demonstrated that two individuals from Shang and Zhou dynasties in Guangdong, China, suffered head injuries and decapitation. Archaeological evidence together with historical accounts suggests the human-induced extinction of this unique crocodylian only a few hundred years ago.

## 1. Introduction

Extant crocodylians are large semi-aquatic predators represented by approximately 30 species in the tropics and subtropics [1]. Many of the living species are currently under threat of extinction [2,3], yet few others experienced population recovery in recent decades [4–6]. Despite the low taxonomic and morphological diversity of extant species, fossil crocodylians exhibit unprecedented craniodental and postcranial morphologies, such as duck-like and extremely long snouts, ziphodont teeth and hoof-like unguals [7,8]. Moreover, morphometric analyses demonstrated that fossil species took currently unoccupied regions of skeletal morphospace, and crown group crocodylians underwent multiple disparity peaks during the Cenozoic [9–11].

Gavialoidea is a group of slender-snouted crocodylians including the living ecomorphological end-member, the Indian gharial (*Gavialis gangeticus*), which is characterized by a slender snout and short limbs [10,12]. Although this group is of importance to understand the evolutionary process of snout shape and associated skeletal morphologies [7,13], the clade position and membership, and relationships among clade members are still disputed [14–17]. The debate stems from the different phylogenetic positions of the Indian gharial in molecular and morphological trees—the Indian gharial is the extant sister taxon to the Malayan gharial (*Tomistoma schlegelii*) in molecular trees [18–22], whereas it is an outgroup to all other extant crocodylians in morphological trees [16,23–26], except few recent ones that are consistent with the molecular tree [17,27]. Contrasting phylogenetic hypotheses based on molecular and morphological data confounded interpretation of cranial and postcranial evolution in crocodylians [10,14,15]. Although improved analytical protocols are required, the most important key to resolving the molecular–morphological phylogenetic conflict is sampling of new fossil taxa [16,19].

Here, we report a new, exceptionally well preserved crocodylian from the Bronze Age of southern China. Although the new Chinese crocodylian superficially resembles tomistomines, it shows a wealth of gavialine features including a potentially sexually selected vocal structure, reducing the morphological gap between gavialines and tomistomines and providing insights into sexual selection on acoustic signalling in crocodylians. It also represents one of the most compelling examples of the human-mediated reptile extinction in the late Quaternary. Chop marks left on the skeletons of two Bronze-age specimens together with historical accounts suggest that the human–crocodylian conflict had lasted in southern China from the Bronze Age until a few hundred years ago when this unique species finally became extinct.

## 2. Methods

### (a) Body length estimation

Snout–vent length (SVL) and total length (TL) of the holotype (XM 12-1558) and a paratype (SM E1623) of *Hanyusuchus sinensis* gen. et sp. nov. were estimated from the sum of the presacral centrum lengths and the sum of the cervical centrum lengths, respectively, using the equations in Iijima and Kubo [28]. Body lengths of the remaining paratypes without postcrania (XM 12-1557 and SM S01812) were estimated based on the skull–body length proportions in the holotype.

### (b) Accelerator mass spectrometry radiocarbon dating of bones

Lower jaw and rib fragments in the holotype (XM 12-1558) and paratypes (XM 12-1557; SM E1623) of *H. sinensis* were used for the dating. Collagen was extracted from the organic remains and purified using the gelatinization method [29]. Weight percentages and stable isotope ratios of carbon and nitrogen were measured using the Thermo Fisher Scientifics EA-IRMS system and compared with standard materials for calibration [30,31]. Following the routine CO_2_ trapping and graphitization [32], the ^14^C/^12^C ratio of graphite was measured with the AMS system in the Laboratory of Radiocarbon Dating, The University of Tokyo, Japan. *δ*^13^C measured in conjunction with the ^14^C analysis was used for the isotopic fractionation correction [33]. Calibration of radiocarbon age determinations was done using the IntCal20 curve [34] in OxCal v. 4.2 [35]. Calibrated ages (in cal BP) were reported with 1-sigma error.

### (c) Cutmark analysis

A paratype skull (XM 12-1557) of *H. sinensis* preserves over a dozen chop marks on the skull table and the occipital condyle. Orientations of chop marks on the skull table with respect to the mediolateral axis of the skull were measured based on a photo image. A rose diagram was generated for showing the distribution of chop mark orientations. Biaxial orientation data were converted to unimodal circular data (0–2π radians), using the equation *α*_adj_ = 2*α*(mod 2π), where *α* and *α*_adj_ are original and adjusted orientations in radian, respectively [36]. Uniformity of orientation distribution was examined with the Rayleigh test, using the R package circular [37,38]. For enhanced visualization and measurements, silicon moulds of selected cutmarks were scanned using the Olympus LEXT OLS 4100 confocal laser microscope in the Light Imaging Facility, Clemson University, USA. Captured surface images were inverted in the operating software to match the real cutmark contour, and measurements were taken digitally.

### (d) Phylogenetic analyses

To assess the phylogenetic relationship of *H. sinensis*, we performed Bayesian and maximum-parsimony analyses using the revised data matrix (77 taxa and 254 discrete morphological characters) of Iijima and Kobayashi [15], which was largely based on earlier works (e.g. refs. [23,25,26,39]). Analyses were conducted with and without a backbone topological constraint of extant and subfossil taxa based on a molecular tree [22]. *Bernissartia fagesii* was designated as an outgroup and 15 characters were ordered. Bayesian analyses were executed in MrBayes v. 3.2.7 [40]. Markov chain Monte Carlo analyses were run with four chains in each of the two runs for 10 million generations, sampling every 10 000 generations, which resulted in 1000 samples per run. Maximum-parsimony analyses were executed in TNT v. 1.5 [41,42] without or with extended implied weighting (*k* = 3 or 12). Heuristic searches were run with 10 000 random-addition-sequence replicates. See electronic supplementary material, part III for extended methodology of phylogenetic analyses.

## 3. Results

### (a) Systematics

Crocodylia Gmelin, 1789 (*sensu* Benton and Clark, 1988 (ref. [43])).

Gavialidae Adams, 1854 (*sensu* Brochu, 2003 (ref. [44]), molecular context).

*Hanyusuchus sinensis* gen. et sp. nov.

#### (i) Etymology

The generic name after Han Yu (a Chinese government official and poet during the Tang dynasty) + suchus (Latin for Greek soûkhos, the crocodile god Sobek), and the specific epithet after sinae (Latin for China) + ensis (Latin for ‘from’).

#### (ii) Holotype

XM 12-1558, skull, lower jaw and partial postcrania from Dalincun, Tangxiazhen, Pengjiang District, Jiangmen, Guangdong Province (3327 ± 53 cal BP (1 s.d.)) (electronic supplementary material, figure S1).

#### (iii) Paratypes

XM 12-1557, skull from Shitoucun, Tangxiazhen, Pengjiang District, Jiangmen, Guangdong Province (3297 ± 48 cal BP (1 s.d.)); SM E1623, skull, lower jaw and partial postcrania from Longyancun, Leiliuzhen, Shunde District, Foshan, Guangdong Province (2942 ± 55 cal BP (1 s.d.)); SM S01812, skull from Sijicun, Ronggui, Shunde District, Foshan, Guangdong Province (radiocarbon age unknown) (electronic supplementary material, figure S1).

#### (iv) Distribution

**Figure 1.**
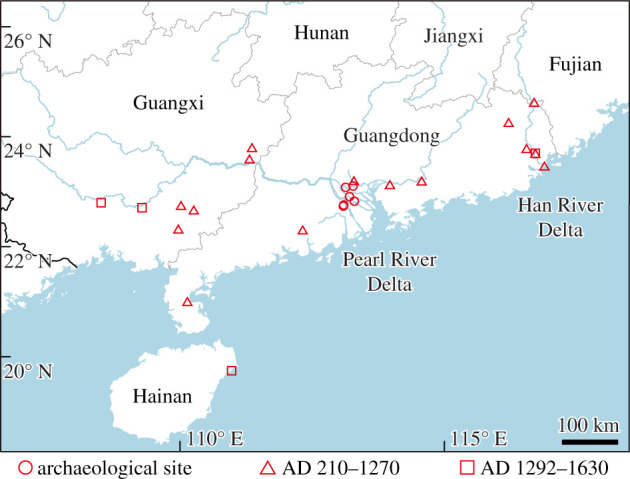
Historical distribution of *Hanyusuchus sinensis*, gen. et sp. nov. in Southern China (electronic supplementary material, figure S1 and tables S1 and S2 for details). (Online version in colour.)

Archaeological records and historical literature revealed the past occurrences of *H. sinensis* across Fujian, Guangdong, Guangxi, and Hainan provinces in southeastern China from the late fourth millennium BC to the mid second millennium AD (figure 1; electronic supplementary material, figure S1 and tables S1 and S2).

#### (v) Diagnosis

**Figure 2.**
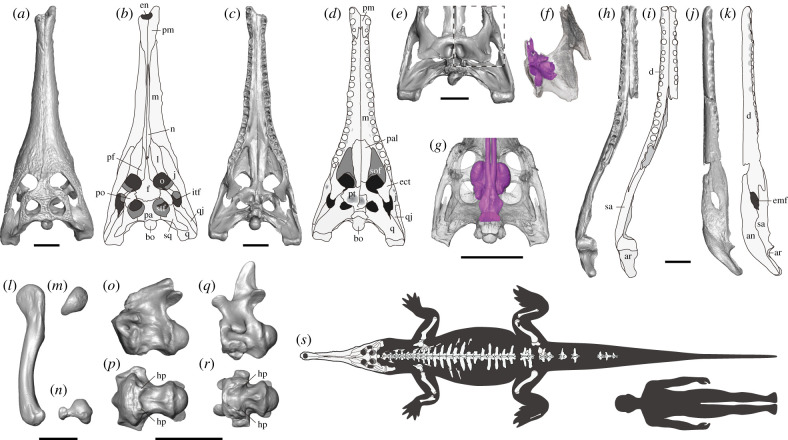
Anatomy of *Hanyusuchus sinensis*, gen. et sp. nov. from the Bronze Age of southern China. (*a*–*d*) Holotype skull (XM 12-1558) in dorsal (*a,b*) and ventral (*c,d*) views. (*e*) Posterior skull of a paratype (XM 12-1557) in ventral view. (*f*) Three-dimensional reconstruction of the boxed part in (*e*), highlighting the pterygoid bulla (purple volume). (*g*) Three-dimensional reconstruction of the pterygoid bullae and nasopharyngeal duct (purple volume) in *Gavialis gangeticus* (UF 118998). (*h*–*k*) Holotype mandible (XM 12-1558) in dorsal (*h,i*) and left lateral (*j,k*) views. (*l*–*n*) Left femur of the holotype (XM 12-1558) in lateral (*l*), proximal (*m*) and distal (*n*) views. (*o,p*) Axis of a paratype (SM E1623) in left lateral (*o*) and ventral (*p*) views. (*q,r*) Third cervical vertebra of the holotype (XM 12-1558) in left lateral (*q*) and ventral (*r*) views. (*s*) Composite reconstruction of *H. sinensis* scaled to the holotype (XM 12-1558) compared with a human (1.8 m height). an, angular; ar, articular; bo, basioccipital; d, dentary; ect, ectopterygoid; emf, external mandibular fenestra; en, external naris; f, frontal; hp, hypapophysis; itf, infratemporal fenestra; j, jugal; l, lacrimal; m, maxilla; n, nasal; o, orbit; pa, parietal, pal, palatine; pf, prefrontal; pm, premaxilla; po, postorbital; pt, pterygoid, q, quadrate; qj, quadratojugal; sa, surangular; sof, soborbital fenestra; sq, squamosal; stf, supratemporal fenestra. Scale bars are 10 cm. (Online version in colour.)

A large slender-snouted crocodylian with five premaxillary, 16 maxillary and 18 dentary teeth; seventh maxillary tooth largest in the anterior-mid maxilla; medial wall of the last three maxillary alveoli within the suborbital fenestrae swollen; dorsal half of the prefrontal pillar narrow anteroposteriorly, and the medial process of the pillar dorsoventrally tall and anteroposteriorly short; interfenestral bar wider than one half of the interorbital distance; postorbital and squamosal parts of the skull table slope laterally; a shallow fossa extending posteriorly from the supratemporal fenestra onto the dorsal squamosal surface (autapomorphy); a pair of deep depressions anterior to the internal choana associated with the expansion of the posterior chamber of the pterygoid bulla; exoccipital sending a robust process ventrally to the basioccipital tubera; a pair of knob-like hypapophyses on the ventral surface of axial and third cervical centra; anterior margin of dorsal midline osteoderms with anterior process; reduced medial condyle of the femur (figure 2*a–r*; see electronic supplementary material, part I and II and figures S2–S21 for full description and comparisons, and electronic supplementary material, tables S3–S5 for measurements).

#### (vi) Maturity and body size

The holotype (XM 12-1558) and a paratype (SM E1623) show closed neurocentral sutures in precaudal vertebrae, indicating sexual maturity [28,45,46], although their sexes are unknown. Body length (SVL and TL) estimates are 2.83 m SVL and 5.43 m TL for XM 12-1558 (figure 2*s*), 3.23 m SVL and 6.19 m TL for XM 12-1557, 2.88 m SVL and 5.57 m TL for SM E1623 and 3.09 m SVL and 5.97 m TL for SM S01812.

### (b) Cutmarks

**Figure 3.**
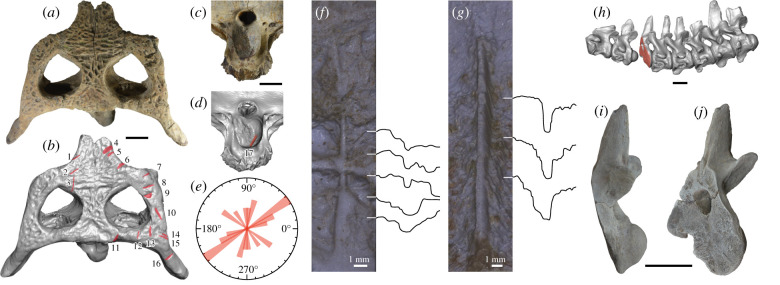
Chop marks left on *Hanyusuchus sinensis*, gen. et sp. nov. from the Bronze Age of southern China. (*a*–*d*) Seventeen chop marks on the skull table to periorbital region (*a,b*) and the occipital condyle (*c,d*) in a paratype (XM 12-1557). (*e*) Orientations of chop marks on the skull table with respect to the mediolateral axis of a paratype skull (XM 12-1557). (*f,g*) Close-up of chop marks no. 3 (*f*) and no. 17 (*g*) in a paratype skull (XM 12-1557). (*h*) Cervical vertebrae in a paratype (SM E1623) highlighting the cut surface of the fourth cervical vertebra. (*i,j*) Posterior half of the bisected fourth cervical vertebra in a paratype (SM E1623) in lateral (*i*) and anterolateral (*j*) views. Scale bars for *a*–*d*, *h–j* are 5 cm. (Online version in colour.)

Seventeen chop marks, 16 on the skull table to periorbital region and one on the occipital condyle were found in a paratype (XM 12-1557: figure 3*a*–*d*; electronic supplementary material, figure S22). Chop marks on the skull table to periorbital region are mostly distributed on its right side. Although some chop marks show similar orientations, the Rayleigh test did not reject uniform distribution of chop mark orientations (*z* = 2.181, *p* = 0.112) (figure 3*e*). All the chop marks have straight or nearly straight edges. Vertical chop marks (no. 1–3, 7, and 17) are narrow and deep, showing the smooth floor. The two longest vertical chop marks on the anterior skull table (no. 3) and occipital condyle (no. 17) are 16.3 mm and 19.2 mm in length, respectively. Their maximum depths and breadths where both cutting edges are clearly defined are 0.7 mm and 0.6 mm, respectively in no. 3 and 3.2 mm and 0.8 mm, respectively in no. 17 (figure 3*f,g*). Obliquely angled chop marks (no. 4–6 and 8–16) often have a smooth and flat kerf wall and an irregularly fractured wall on the other side that are characteristics of metal chop marks [47,48]. The kerf wall widths measured perpendicular to the bottom grooves are approximately 6–7 mm for no. 4, 10 and 11. Similar orientations of closely spaced chop marks (no. 4–9) indicate multiple chops by the same person. Because the smooth kerf walls of these chop marks face posterodorsally, a right-handed person might chop the skull from its right side. Chop marks oriented in different directions (no. 3 and 10–13) could be made by the same person from different positions or by other people. A prominent chop mark was also found in the fourth cervical vertebra of a different paratype (SM E1623: figure 3*h*–*j*; electronic supplementary material, figure S23), as briefly mentioned by Zeng [49]. The entire vertebra was bisected obliquely in a single blow. The cut surface (110 mm height and 79 mm width) is flat and clean, exposing the cancellous bone tissues. The cutting edge is sharp and clearly defined on the left side and slightly fractured on the right side, suggesting the chopping from the left to right side.

### (c) Phylogenetic analyses

**Figure 4.**
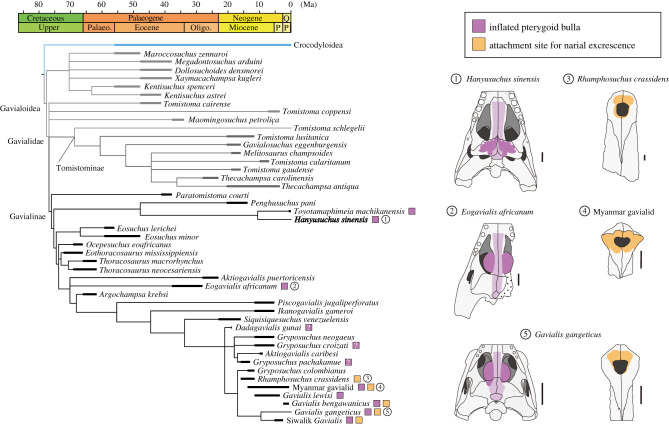
Gavialoid phylogeny and the evolution of the acoustic apparatus. Phylogenetic relationships were obtained from Bayesian analysis with a backbone constraint of extant and subfossil taxa based on a molecular tree [22]. Shared branch lengths were divided equally using the R package palaeotree [38,50], and branch colours correspond to those in figure 5. Phylogenetic distribution of the inflated pterygoid bulla and the attachment site for the narial excrescence are based on first-hand observation and literature [51–59]. Illustration source: *Eogavialis africanum* (Senckenberg Museum specimen [56]: pathway of the nasopharyngeal duct estimated); *G. gangeticus* (BMNH 2005.1601; UF 118998); *Hanyusuchus sinensis* (XM 12-1557; XM 12-1558); Myanmar gavialid (MZKB F1160) and *Rhamphosuchus crassidens* (BMNH 39802). All scale bars are 5 cm. (Online version in colour.)

A 50% majority rule consensus tree from the Bayesian analysis with a topological constraint of extant and subfossil taxa based on the molecular hypothesis is shown in figure 4 (also see electronic supplementary material, figure S24*a*). The constrained Bayesian tree recovered the clade of *Penghusuchus pani*, *Toyotamaphimeia machikanensis* and *H. sinensis* as sister to *Eosuchus* and more derived gavialines as in constrained parsimonious trees (electronic supplementary material, figure S25 and table S6). The unconstrained Bayesian tree recovered Alligatoroidea as an outgroup of Gavialoidea + Crocodyloidea (electronic supplementary material, figure S24*b*), unlike the unconstrained parsimonious trees that recovered Alligatoroidea as sister to Crocodyloidea (electronic supplementary material, part IV, figure S26 and table S6).

## 4. Discussion

### (a) A new Chinese crocodylian as a key taxon to resolving the molecular–morphological phylogenetic conflict

*H. sinensis* is an intermediate taxon that reduces the morphological gap between two lineages leading to Indian and Malayan gharials (gavialines and tomistomines in the molecular context: ref. [44]). Previously, two extant gharials were either distantly placed in the majority of the morphological trees ([14,23,26]; but see [17,27] that indicated otherwise) or formed an extant sister group in molecular or combined morphological + molecular trees [14,15,19,60]. Recent work demonstrated the presence of gavialine features in post-Palaeogene crocodylians from East Asia (*P. pani* and *T. machikanensis*) that were previously considered as tomistomines, rendering support for the molecular tree [15]. Despite the superficial resemblance to tomistomines, *H. sinensis* shares many of those gavialine features, such as (i) exoccipital sending a robust and anteroposteriorly wide descending process to the basioccipital tubera (electronic supplementary material, figure S7); (ii) splenial symphysis wide V-shape in dorsal view (figure 2*h,i*); (iii) forked axial hypapophyses (figure 2*p*); (iv) absence of keel-like hypapophysis in the third cervical vertebra (figure 2*r*); (v) inter-zygapophyseal widths narrow in dorsal vertebrae (electronic supplementary material, figures S13 and S14) and (vi) dorsal or pelvic midline osteoderms bearing anterior process (electronic supplementary material, figure S21).

Exquisite preservation of *H. sinensis* allowed observation of many more gavialine features. The dorsal half of the prefrontal pillar is narrow anteroposteriorly, and the medial process is dorsoventrally tall and anteroposteriorly short in *H. sinensis* (XM 12-1558; SM S01812: electronic supplementary material, figure S8*c*–*f*), resembling stem and basal crocodylians and gavialines [61,62]. The postorbital and squamosal parts of the skull table slope laterally in *H. sinensis* (XM 12-1557; XM 12-1558; SM S01812; electronic supplementary material, figure S2*c*,*f*,*i*) as in stem crocodylians, gavialines and a few tomistomines [62,63]. The lateral sloping of the skull table in *H. sinensis* is moderate compared with that in gavialines, but the sloping is obvious in occipital view. Additionally, the medial condyle of the femur is reduced and considerably smaller than the lateral condyle in distal view in *H. sinensis* (XM 12-1558; figure 2*n*), which is shared with *G. gangeticus* (AMNH 88316; UF 70592; UF 118998) and *G. bengawanicus* [51]. In other gavialoids including *Eosuchus minor* (USNM 355967) and *Maomingosuchus petrolica* (IVPP V5015), anteroposterior lengths of the medial and lateral condyles are subequal, or the medial condyle is only slightly shorter than the lateral one in distal view.

**Figure 5.**
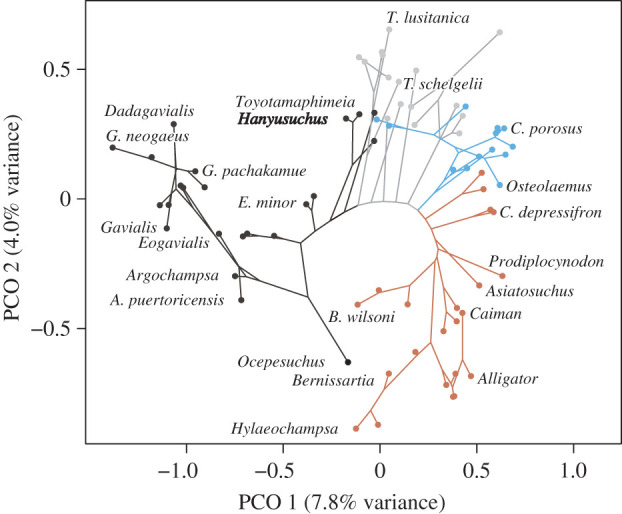
Phylomorphospace based on the principal coordinate analysis of the morphological distance matrix converted from the cladistic character dataset (77 taxa and 254 characters) and the constrained Bayesian tree. Branch colours are black for Gavialinae, grey for non-gavialine gavialoids, blue for Crocodyloidea and brown for non-Longirostres crocodylians (figure 4; electronic supplementary material, figure S24*a*). (Online version in colour.)

Although our phylogenetic trees without a topological constraint are overall consistent with previous morphological trees, those with a constraint on extant and subfossil taxa placed post-Palaeogene crocodylians from East Asia (*P. pani*, *T. machikanensis* and *H. sinensis*) at basal Gavialinae near the split of gavialines and tomistomines (figure 4). A mosaic of gavialine and tomistomine features in these East Asian taxa explains their basal gavialine status in constrained morphological trees [15,17]. A phylomorphospace based on the cladistic character dataset corroborates the notion that three East Asian crocodylians are intermediate taxa linking gavialines and non-gavialine gavialoids (figure 5; see electronic supplementary material, part III for methodology). These East Asian taxa occupy the edge of gavialine morphospace and are partially overlapped with non-gavialine gavialoids.

### (b) Implication for sexual selection on acoustic signalling in crocodylians

*H. sinensis* exhibits a cranial feature that is relevant to acoustic function unique to mature male Indian gharials. Multiple sinuses that occupy the pterygoid in *H. sinensis* (figure 2*f*; electronic supplementary material, figure S6*c*) would be homologous to the posterior chamber of the pterygoid bulla, which is confined to the anterior part of the pterygoid in the Indian gharial (figure 2*g*; refs. [52,64]). Although the dorsal surface of the palatine is broken in *H. sinensis*, it might also have the anterior chamber of the pterygoid bulla as in the Indian gharial. The anterior and posterior chambers of the pterygoid bulla, the closed-end chambers, are connected to the nasopharyngeal duct and would change harmonics of the sound, depending on the chamber lengths [52]. In the Indian gharial, inflation of the bulla during ontogeny is coupled with development of the narial excrescence, a soft tissue surrounding the osteological external naris, which houses the dorsoposterior extension of the nasal cavity [52]. Elongation of the nasal cavity would reduce dispersion of formant, the resonance frequency of the vocal tract—the hypothesis supported by change in formant frequencies yet near constant fundamental frequencies of the source signal during the experimental alteration of vocal tract lengths in Chinese alligators [65] and through ontogeny in American alligators [66]. Because vocal tract lengths are physically constrained and correlated with body size in many mammals and birds and a few crocodylians examined thus far, formants were considered as honest cues of body size for intraspecific communication in those taxa [66–68]. The narial excrescence that elongates the vocal tract likely evolved to exaggerate size in the Indian gharial, which would be functionally analogous to the cranial crest enclosing the elongated nasal cavity in lambeosaurine hadrosaurs [69] and the elongated trachea in some birds [70]. Presumably, the cost of vocal tract elongation (e.g. increased drag during underwater head sweeping) was smaller than the advantage of size exaggeration in male Indian gharials.

Potential evidence of sexual selection on the acoustic size exaggerator (narial excrescence) and sound modifier (pterygoid bulla) has been known in extinct gavialoids (figure 4). Perinarial depressions in ‘tomistomines’ *Rhamphosuchus crassidens* and ‘*Gavialis’ pachyrhynchus* [52,53,71] and a pair of rugose-tipped protuberances on both sides of the naris in a Myanmar gavialid and *G. bengawanicus* [51,54] were considered as the attachment sites for the narial excrescence. Additionally, the presence of the inflated anterior chamber of the pterygoid bulla was confirmed in *Eogavialis africanum*, *G. lewisi* and *G. bengawanicus* [51,55,56,72] and suggested in *Dadagavialis gunai*, *Gryposuchus croizati* and *G. pachakamue* [57–59]. Furthermore, *H. sinensis* shows a novel expansion of the posterior chamber of the bulla, increasing the bulla morphological diversity. The expanded posterior chamber of the bulla is associated with the development of a pair of deep depressions anterior to the internal choana in *H. sinensis* (XM 12-1557; XM 12-1558; SM S01812: electronic supplementary material, figure S6*b*). Similarly large anterior choanal depressions are known in *G. lewisi* [55,73], implying the existence of the expanded posterior chamber in this species.

Assuming that the narial excrescence and the inflated pterygoid bulla are mature male-only throughout the crocodylian evolution, our phylogenetic tree that was forced to fit the molecular hypothesis indicates the repeated evolution of sexually selected acoustic apparatus within Gavialinae, a slender-snouted group of crocodylians (figure 4). The Indian gharial and fossil gavialines with the possible narial excrescence are large to giant taxa [2,51,53,74], consistent with the acoustic size exaggeration hypothesis that posits selection for larger body size precedes sexual selection on the size exaggerator [70]. The function of modified harmonics through the pterygoid bulla is ambiguous, but its presence only in slender-snouted gavialines may suggest the auxiliary role in size deception, as selection for the longer snout, thus the longer vocal tract, augments acoustic size exaggeration. Conceivably, extinct gavialines might rely more on acoustic than visual size cues, as the extant gavialine Indian gharial does not use head oblique tail arched posture [75], an honest visual body size signal [76].

### (c) Human-induced extinction of a large crocodylian in southern China

*H. sinensis* is of archaeological significance as it provides direct evidence of human–animal interaction in ancient China. Over a dozen of chop marks on the skull table to periorbital region in XM 12-1557 (figure 3*a*,*b*) imply an intention of killing this individual by inflicting wounds on the head, while a chop mark on the occipital condyle (figure 3*c,d*) might be the result of postmortem dismemberment. Similarly, a large chop mark on the fourth cervical vertebra in SM E1623 (figure 3*h*–*j*) could result from decapitation. Given the anterior inclination of the cut surface, the executor chopped down the neck of this individual diagonally in the left anterodorsal to right posteroventral direction, probably aiming at the gap between postoccipital and nuchal osteoderms. Decapitation of this large individual likely required multiple chops to sever soft tissues around the vertebra, although the bone appears to be bisected in a single blow.

The parallel-walled, deep vertical chop marks in the skull (XM 12-1557; figure 3*f,g*) and a large cut surface of the vertebra (SM E1623; figure 3*i,j*) support the use of heavy metal implements. Two chop mark bearing specimens from Guangdong are dated to Shang and Zhou dynasties (14–10th century BC) in the Chinese Bronze age, during which bronze axes were symbols of monarchical power and religious activities [77]. Although the appearance of bronze cultures in southeastern China lagged behind Yellow and Yangtze valley regions, multiple Shang dynasty culture sites in Guangdong yielded bronze tools, weapons, and casting moulds, indicating the presence of local bronze metallurgy [78–80]. These suggest that bronze weapons like axes as potential implements used for chopping the crocodylians.

Historical Chinese literature depicted human–crocodylian conflict in southeastern China from the early first to mid second millennium AD. The ancient Chinese crocodylian, most likely *H. sinensis*, was described as a huge aquatic animal with a long snout and sharp teeth, which often attacked people and livestock [81] (electronic supplementary material, table S1). To remove and exterminate crocodylians, government officials in Tang, Song and Ming dynasties (9–15th century AD) resorted to sacrificial rituals and forces in the Han River valley, eastern Guangdong [81,82] (electronic supplementary material, table S1). The ancient Cantonese hatred of crocodylians had lasted for at least three millennia since the Shang dynasty, as two chop mark bearing specimens from Shang and Zhou dynasties were discovered in the Pearl River delta, Guangdong.

The extinction of *H. sinensis* was supposedly caused by human-induced habitat degradation and loss, along with killing as evidenced by archaeological records and ancient literature. Historically, before the mid second millennium AD, *H. sinensis* was distributed along the major river systems across Guangxi, Guangdong and Fujian (figure 1; electronic supplementary material, figure S1). The range of *H. sinensis* would be progressively contracted during the past two millennia, as hotspots of the population associated with intensive agricultural activities emerged in southern China [83,84]. These population and agricultural dynamics appear to be closely tied to the range contraction and extirpation of Chinese mammalian megafauna (elephants, rhinoceroses, tigers and bears) [84]. Although climate changes also played roles in the megafauna extinction in China and other zoogeographical regions [85–87], it would not be the major driver of the demise of *H. sinensis*, because they survived until a few hundred years ago.

## Data Availability

Data supporting the results of this study (full description of *H. sinensis*, morphological comparisons, extended methodology, results of maximum-parsimony analyses, supplementary figures and tables, MrBayes and TNT files used in the phylogenetic analyses, and data and codes used for generating phylomorphospace) are provided in electronic supplementary material [88].
